# Evolution of focal nodular hyperplasia during long-term ultrasound follow-up: results from a single-center study

**DOI:** 10.1007/s40477-025-01010-1

**Published:** 2025-03-25

**Authors:** Mattia Paratore, Matteo Garcovich, Annamaria Amodeo, Francesca Fianchi, Francesco Santopaolo, Francesca Romana Ponziani, Maria Assunta Zocco, Antonio Gasbarrini, Laura Riccardi, Maurizio Pompili

**Affiliations:** 1 Diagnostic and Interventional Ultrasound Unit, CEMAD Digestive Disease Center, Catholic University of the Sacred Heart, Fondazione Policlinico Universitario Gemelli IRCCS, Rome, Italy; 2 Internal Medicine and Liver Transplantation Unit, Catholic University of Sacred Heart, Catholic University of the Sacred Heart, Fondazione Policlinico Universitario Gemelli IRCCS, Rome, Italy; 3 Internal Medicine and Gastroenterology Unit, CEMAD Digestive Disease Center, Catholic University of the Sacred Heart, Fondazione Policlinico Universitario Gemelli IRCCS, Rome, Italy; 4 Translational Medicine and Surgery Department, Università Cattolica del Sacro Cuore, Rome, Italy

**Keywords:** Focal nodular hyperplasia, Benign liver tumours, Liver ultrasound, Ultrasound follow-up

## Abstract

**Purpose:**

To examine the natural history of focal nodular hyperplasia (FNH) lesions through long-term ultrasound (US) follow-up and assess the relationship between clinical characteristics and size changes.

**Methods:**

We retrospectively enrolled 55 patients diagnosed with FNH who were followed with ultrasound for at least 24 months. A total of 94 FNH nodules were included in the final analysis. A significant change in size was defined as an increase or decrease of 0.5 cm or more, and nodules were classified as increased, decreased or stable. Additionally, we analyzed the association between clinical data and changes in nodule size.

**Results:**

The mean follow-up duration between the initial US examination at diagnosis and the last available examination was 58.3 ± 33.5 months (range: 24.2–186.6). The majority of nodules remained stable (47.9%) or decreased in size (35.1%), while a small proportion of nodules disappeared (11.7%) and only 7.3% showed an increase in size. No significant association was found between size variation and factors such as oral contraceptive use, pregnancy, BMI or follow-up duration.

**Conclusion:**

Changes in the size of FNHs during follow-up are relatively common, with most lesions remaining stable or undergoing regression or disappearance over time. These size variations do not appear to be influenced by hormonal factors or other clinical characteristics.

## Introduction

Focal nodular hyperplasia (FNH) is the second most common benign liver tumor, with an estimated prevalence in general population of 0.2–3% [1].

According to the most widely accepted pathogenetic theory, FNH is caused by a localized arterial dystrophy, which leads to regeneration and polyclonal expansion of hepatocytes and deposition of fibrotic tissue [2]. This hypothesis is supported by research demonstrating a change in the mRNA expression levels of angiopoietin genes (ANGPT1 and ANGPT2) involved in vascular development in all FNH samples examined [3]. A recent study shows that the majority of FNH are genetically stable and identifies FNH-specific SOST-expressing endothelial cells and PDGFRB + fibroblasts that may contribute to fibrogenesis [4].

Generally, FNH occurs as a single nodule often detected in women in childbearing age who undergo abdominal ultrasound (US) for nonspecific symptoms usually unrelated to the presence of the lesion. FNH manifests as multiple lesions in approximately 20% of patients and is associated with the presence of hepatic angiomas in roughly 20% of cases and with hepatic adenoma in only 3.6% of cases [5, 6]. On US, FNH often appears as a well-defined solid nodule, devoid of a capsule, with a heterogeneous hypo-isoechoic echo-structure and a pathognomonic central fibrous scar in less than 50% of cases [7]. On color Doppler US examination, the most prominent characteristic is the robust radial vascularization with central arterial signal. In 40% of cases, a venous Doppler signal can be detected at the center of the lesion, limiting the differential diagnosis with hepatocellular adenoma [8]. Contrast-enhanced US (CEUS) reveals a centrifugal hypervascular pattern of contrast enhancement with iso- hyperenhancement in portal and late phase and may be helpful to detect the central scar missed on MRI in nodules smaller than 3 cm [9–11]. Magnetic resonance imagining (MRI) has higher sensitivity than US and computed tomography (CT) for the diagnosis of FNH, as well as a specificity of almost 100%. The MRI sensitivity is further increased up to 90% using the hepatobiliary contrast agents and then histological diagnosis using liver biopsy is exclusively indicated when imaging is inconclusive and should include glutamine synthase immunological staining [11].

Complications due to FNH are uncommon. Large FNH may rarely cause symptoms due to compression of nearby organs or may be worsened by breakthrough bleeding, especially if it occurs in the subcapsular region, or by portal hypertension if it compresses the hepatic hilum [12–15].

It is now widely accepted that pregnancy and the use of oral contraceptives have no impact on the development of FNH [16]. However, nowadays the benefit of follow-up imaging to monitor FNH is still debated. Based on published evidence, the American College of Gastroenterology (ACG) and the Brazilian Society of Hepatology (SBH) suggest a US follow-up to monitor FNH, while the other available guidelines (European Association for the Study of the Liver, EASL; American Association for the Study of Liver Diseases, AASLD), state that no surveillance is required. The Italian guidelines recommend surveillance only in symptomatic patients or in patients with large lesions located in subcapsular or peri-caval areas [11, 17–20].

The main aim of this study is to investigate the natural history of FNH nodules using long-term US follow up imaging and to evaluate the relationship between clinical features and US dimensional data.

## Methods

A retrospective analysis of FNH patients consecutively evaluated from 2010 to 2022 at the Diagnostic and Interventistic Ultrasound Unit of the Fondazione Policlinico Universitario Agostino Gemelli IRCCS was performed. Patients meeting the following inclusion criteria were considered in the final analysis: established diagnosis of FNH using MRI, CT, CEUS or biopsy; an US examination of the liver following diagnosis (T0); a second US examination performed at least 24 months later (T1) by the same operators (M.P; M.G; L.R) who conducted the T0; both T0 and T1 describing the maximum diameter at T0 and T1 and localization of each FNH nodules. For patients in whom more than one US follow up study was available, we used the data retrieved during the most recent evaluation.

The size variation between T0 and T1 was considered significant if it was equal to or greater than 0.5 cm in absolute terms. Nodules showing a reduction or increase in diameter of at least 0.5 cm were categorized as reduced or increased in size, respectively. Nodules with diameter changes of less than 0.5 cm were classified as stable. Additionally, a percentage-based variation was assessed: nodules with a change of more than 10% in diameter were classified as increased or decreased.

Demographic data (age, sex), anthropometric data (weight, height, BMI), clinical data (assumption of oral contraceptives and pregnancies, symptoms or complication FNH-related) and data relating to the US examination were retrieved from the hospital records.

This study was performed in line with the principles of the Declaration of Helsinki. Approval was granted by the Ethics Committee of Fondazione Policlinico Gemelli (Date 02.08.2023/No 5920). Informed consent was obtained from all individual participants included in the study.

### Statistical analysis

Categorical data were reported as number and percentage while continuous variables were reported as mean ± standard deviation or median and interquartile range according to their distribution. The association between variables was assessed applying Chi-squared tests or Fisher’s exact test. Pearson or Spearman test was used to investigate the correlation between continuous variables according to their distribution. Wilcoxon rank test or paired t-test was used to compare paired data. ANOVA was used to compare continuous unpaired variable. Finally, a p-value ≤ 0.05 was considered statistically significant. GraphPad Prism software version 9.0 was used for data analysis.

## Results

### Demographic characteristics

Demographic, clinical and sonographic characteristics of population are summarized in Table 1. A total of 55 patients, comprising 9 males and 46 females, were included in the study, encompassing 94 nodules. The median age of the cohort was 42.9 years (± 13.8), and the median BMI at diagnosis (T0) was 25.5 kg/m^2^ (± 5.3). Diagnosis of FNH was achieved in 87 nodules via MRI, 1 via CT, 1 via biopsy, and 5 via CEUS. The mean nodule diameter at T0 was 3.3 cm (± 2.0). Among the female patients, 27 of 38 had taken OCP prior to diagnosis, and 24 of 32 had a history of pregnancy. No liver steatosis was detected in 39 patients, while mild, moderate or severe steatosis was established in 6, 5 and 5 patients, respectively. The mean follow-up duration was 58.3 months (± 33.5; range 24.2–186.6) with more than half patients showing a follow-up time longer than 48 months. No patients experienced symptoms or complications during the follow-up period.

**Table 1:** Demographic, clinical and sonographic characteristics of population

N. of patients	55
Sex (M/F)	9/46
Age (Years, SD)	42.9 (± 13.8)
OCP (n/data available)	27/38
Pregnancy (n/data available)	24/32
BMI-T0 (kg/m^2^, SD)	25.5 (± 5.3)
Steatosis (absent/mild/moderate/severe)	27/5/5/4
Hemangioma (%)	13 (23.6%)
Diagnosis of FNH (MRI/CT/ CEUS/biopsy)	39/6/5/5
N. of nodules	94
Diameter-T0 (cm, SD, range)	3.3 (± 2.0, range 0.5–10.6)
Follow-up (months, SD, range)	58.3 (± 33.5, range 24.2–186.6)
N. of patient with Follow-up > 48 months (%)	29 (52.7%)

*CEUS* contrast enhanced ultrasound, *CT* computed tomography, *FNH* focal nodular hyperplasia, *MRI* magnetic resonance imaging, *OCP* oral contraceptive pill

### Dimensional analysis

Eleven nodules whose mean diameter at T0 was 1.4 ± 0.4 cm were not visible at T1 and then were considered to have disappeared. All patients whose nodules disappeared were female and experienced a significant reduction in BMI from T0 to T1 (33.4 vs. 26.3 kg/m^2^, *p* = 0.002). The average follow-up duration was 68 months.

**Fig. 1:**
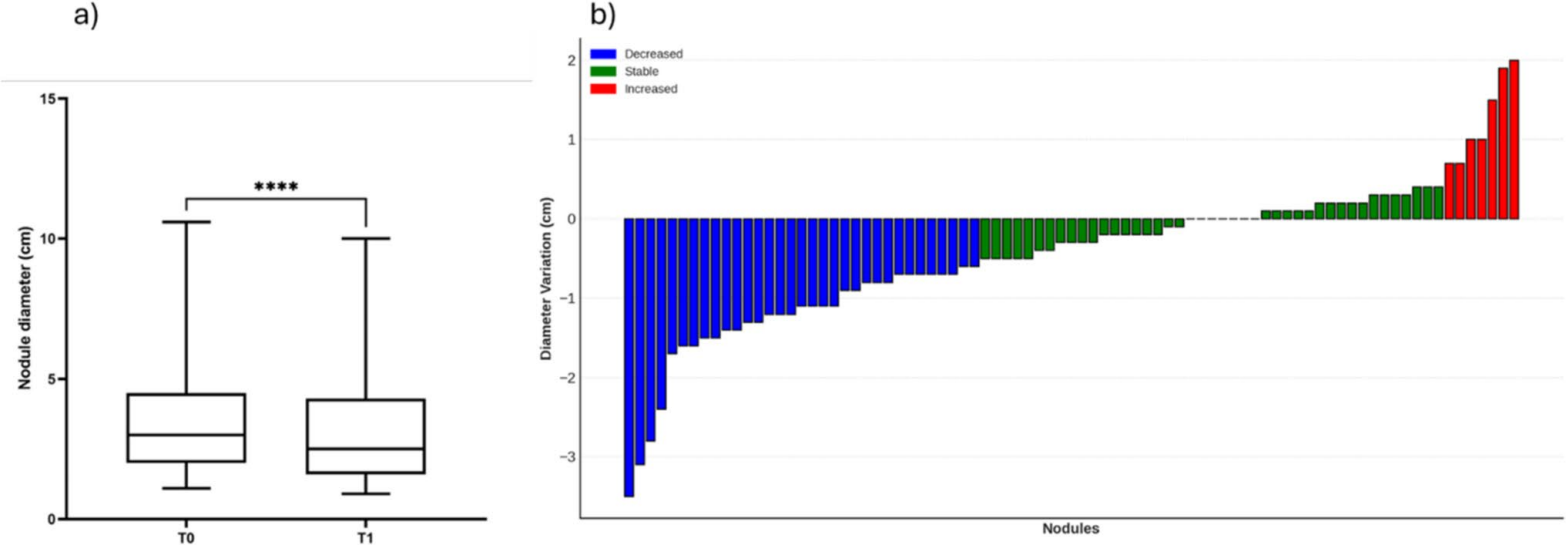
Difference in nodule diameter between T0 and T1 (**a**) and classification according to dimensional variations (**b**)

**Fig. 2:**
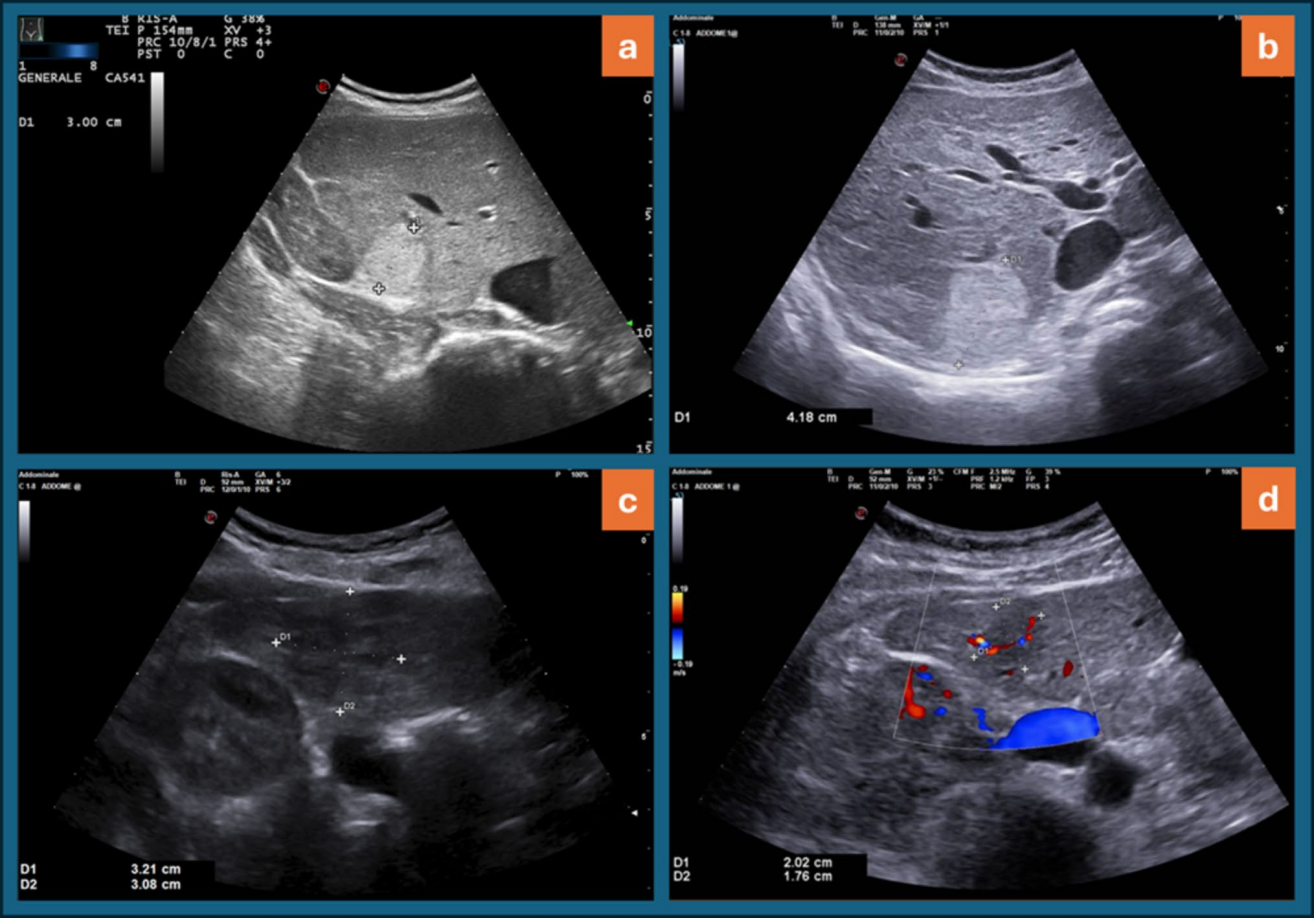
Hyperechoic focal nodular hyperplasia of the VII liver segment increased after 132.9 months (**a**, **b**); hypoechoic focal nodular hyperplasia of the VI liver segment reduced after 75.6 months (**c**, **d**)

Among the remaining 83 nodules detectable on T1, based on the diameter variation of 0.5 cm, 7 nodules were increased, 43 stable and 33 reduced. The mean nodule diameter at T1 was 3.1 cm (± 1.9) and resulted significantly lower than mean diameter at T0 (3.3 ± 2.0 cm, *p* < 0.001) (Fig. 1). Figure 2 provides an example of ultrasound images for increased and reduced FNH nodule after long-term follow-up (Fig. 2).

The average global reduction rate of all nodules was 10.4% corresponding to 0.09 cm/year.

**Fig. 3:**
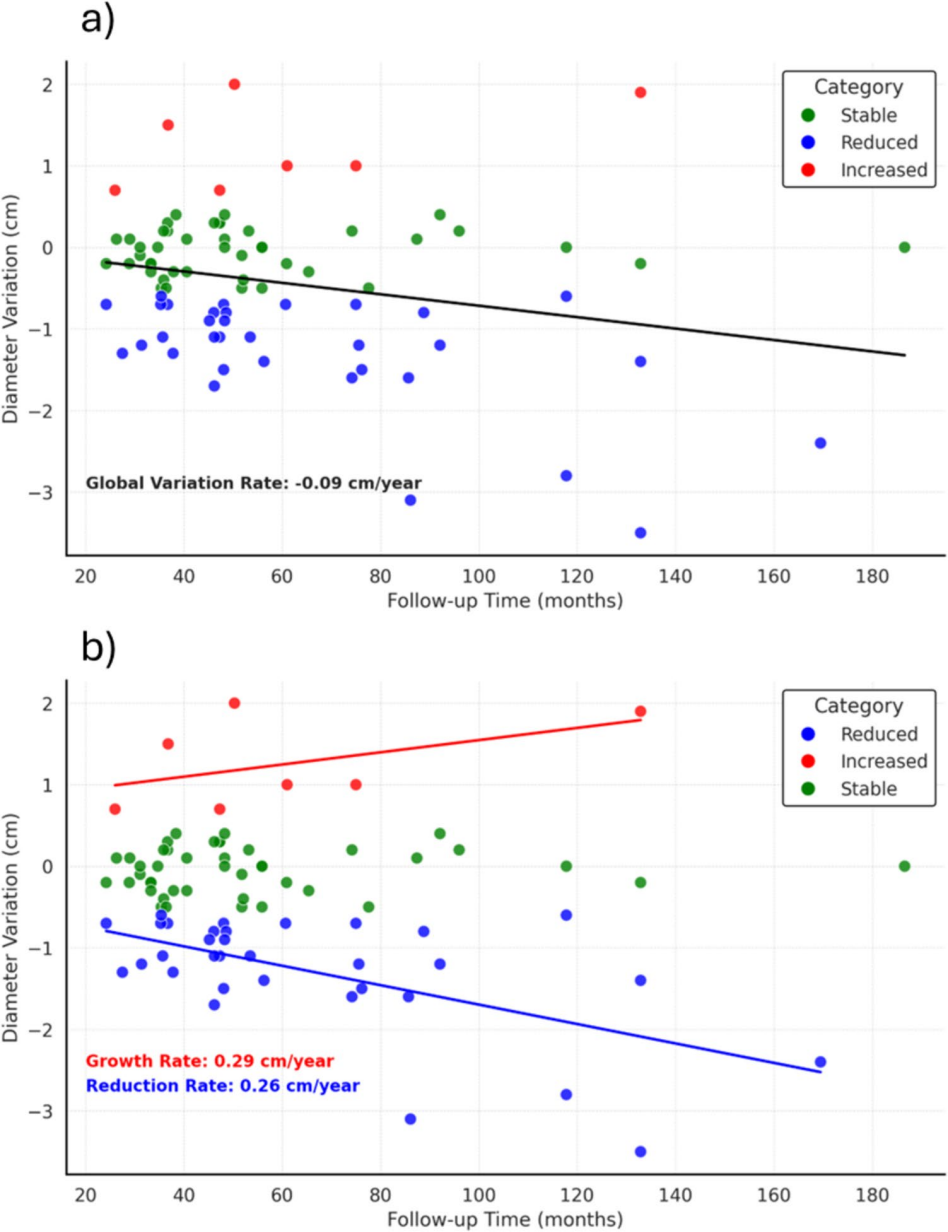
Global (**a**) and categorized (**b**) diameter change rates

The average annual growth rate of the nodules showing an increased size was 0.29 cm/year. The average annual reduction rate of the nodules decreasing in size was 0.26 cm/year (Fig. 3).

**Fig. 4:**
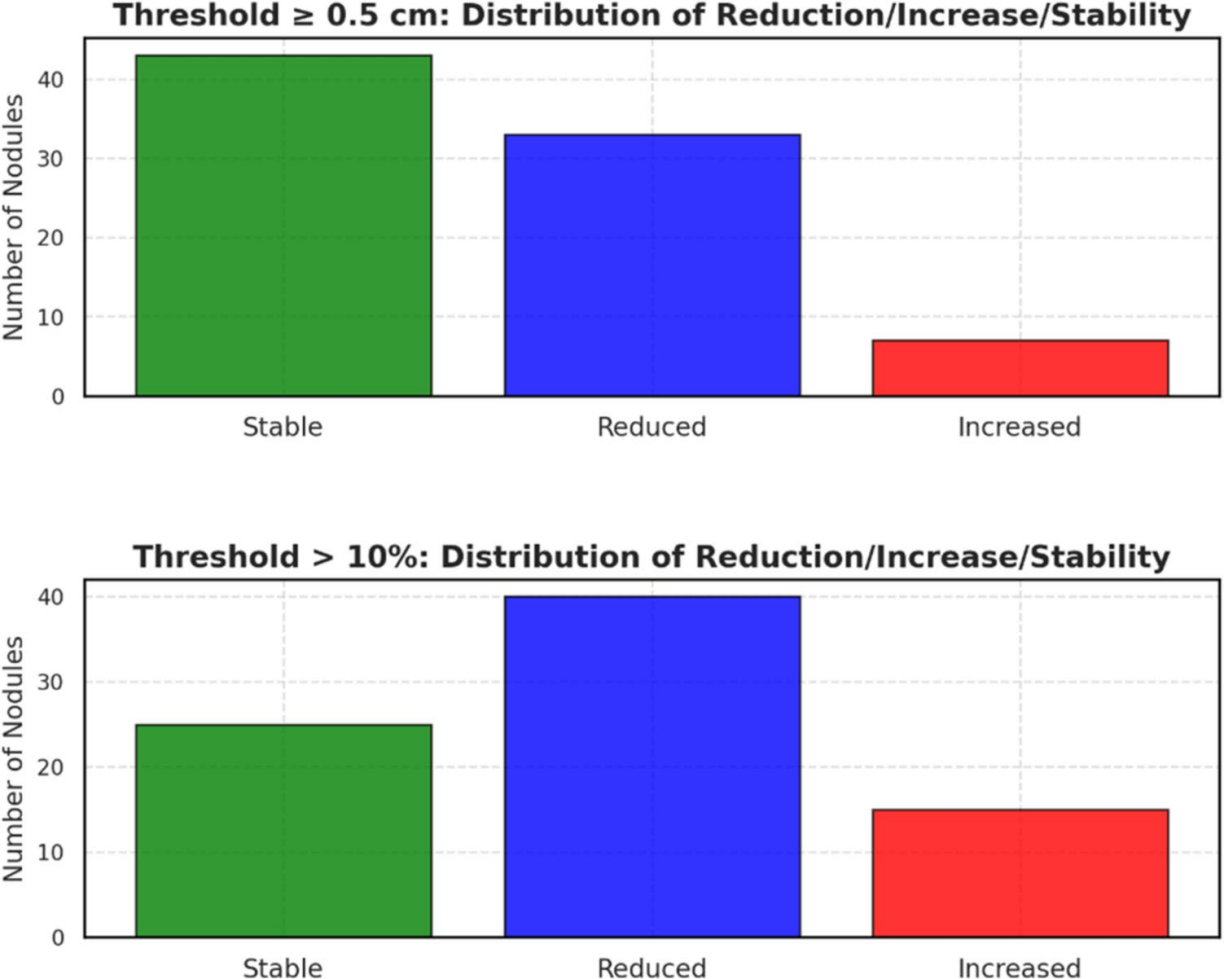
Variation in nodule diameter from T0 to T1: Absolute and Percentage Changes

Comparable results were found when the variation of 10% in the diameter was considered as significant variation (Fig. 4).

Comparing increased and stable/decreased nodules, no significant association was found with sex (*p* = 0.58), OCP use (*p* = 0.99), pregnancy (*p* = 0.83), BMI at T0 (*p* = 0.76) or follow-up length (p = 0.15). No significant correlation was found between the variation in nodule diameter and the length of the follow-up (*ρ* = − 0.15, *p* = 0.19) or variation in BMI (*ρ* = − 0.14, *p* = 0.31).

## Discussion

Our findings highlight that while FNH nodules may change over time, they overwhelmingly remain stable or shrink, with growth being uncommon.

Consistent with existing literature (Table 2), our study cohort predominantly observed stability or reduction in FNH size, and a significant majority (80.9%) of lesions either maintained their size or decreased in size, with only a small proportion (7.4%) demonstrating growth, and the remainder (11.7%) being not detectable over time. In studies using US as a follow-up method, Di Stasi et al. reported that 17 out of 18 nodules remained stable or decreased after nearly 3 years (21), which is similar to the findings of Kuo et al., who observed stability or reduction in 33 out of 34 nodules after a mean follow-up of 42 months.

**Table 2:** FNH and long-term follow-up

References	Study design	FU technique	FNH nodules/patients	Mean FU length	Dimensional evaluation	Results
Di Stasi [21]	Retrospective	US	18/16	33 months	Diameter (variation of 10% more or less)	Disappeared 1/18 Decreased 7/18 Stable 10/18 Increased 0/18
Leconte [23]	Prospective	CT/MRI	18/14	At least 6 months	Area	Stable 6/14 Decreased 2/14
Charny [25]	Retrospective	–	–/42	-	–	Increased 2/42
Kuo [22]	Retrospective	US	34/30	42 months (range 7–95)	Diameter (variation of 10% more or less)	Disappeared 6/34 Decreased 3/34 Stable 24/34 Increased 1/34
Ramirez-Fuentes [24]	Retrospective	MRI	44/30	35 months (range 12–94)	Diameter (variation of 20% more or less)	Decreased 7/44 Stable 35/44 Increased 2/44

*CT* computed tomography, *FNH* focal nodular hyperplasia, *FU* follow-up, *MRI* magnetic resonance imaging

On the other hand, the disappearance of nodules after long-term follow-up is a possible outcome as described also by Di Stasi et al. [21] and Kuo et al. [22], but it could also be related to changes in the US visibility of the hepatic parenchyma, such as the variation in the steatosis grade. In fact, in our cohort, 2 out of 6 patients in whom the nodules disappeared experienced a significant reduction in BMI with the resolution of severe hepatic steatosis detected at baseline. In the remaining cases, the echostructure of the hepatic parenchyma remained unchanged, suggesting a reliable diagnosis of nodules regression. Interestingly, the nodules undetectable at last follow up US study were already small at baseline (mean diameter of the disappeared nodules: 1.3 cm). The stability or the decrease of most FNH nodules has been also shown in case-series based on MRI or CT follow-up [23, 24]. Notably, our study had the longest mean follow-up duration (58 months) and the largest cohort compared to the previous mentioned studies.

No one of the patients enrolled in this retrospective series showed symptoms related to the FNH nodules at the moment of diagnosis and during the follow up. This result supports the recommendation of avoiding any follow up imaging evaluation in asymptomatic patients with FNH reported in the current EASL and AASLD guidelines [19, 20]. We think that imaging periodic reassessment should be limited to the symptomatic patients refusing any treatment of the FNH nodules at the moment of the diagnosis or to the asymptomatic patients reporting symptoms suggesting the occurrence of FNH related complications during the follow up [21].

Regarding demographic and hormonal factors, our findings support a female predominance and early onset, which has suggested in the past literature a potential role of female hormones. While earlier studies have linked the use of oral contraceptives and pregnancy to FNH enlargement, our study has not found a significant association between these factors and dimensional changes, consistent with the findings of Mathieu et al. [16] and in line with the most recent international guidelines [11, 17–20]. In particular, among the 7 patients with increasing nodules size, there were 5 women and no one of them underwent hormonal therapy after the FNH detection.

The main limitation of our study is its retrospective design, which has not permitted a consistent time interval between US evaluations and the acquisition of further clinical data for all patients.

In conclusion, changes in the size of FNHs during US follow-up are relatively common, with most lesions remaining stable or undergoing regression/disappearance over time. These size variations do not appear to be influenced by hormonal factors or other clinical characteristics. Our findings support the indication of avoiding FNH follow-up except for some cases with particular clinical features.

## Data Availability

The data that support the findings of this study are available from the corresponding author, [M.G.], upon reasonable request.
